# Viscoelastic Fingering of Shear-Thinning Drops Impacting on Superhydrophobic Surfaces

**DOI:** 10.1021/acs.langmuir.6c01487

**Published:** 2026-05-07

**Authors:** Diego Díaz, Arivazhagan Geetha Balasubramanian, Kasra Amini, Shervin Bagheri, Outi Tammisola

**Affiliations:** † FLOW and Fluid Physics Laboratory, Department of Engineering Mechanics, 7655 KTH Royal Institute of Technology, 100 44 Stockholm, Sweden; ‡ Swedish e-Science Research Centre, Department of Engineering Mechanics, KTH Royal Institute of Technology, 100 44 Stockholm, Sweden

## Abstract

When water droplets impact solid surfaces at high velocity, they often develop radial protrusions, known as fingering instabilities, that subsequently break up during spreading and retraction, a process termed splashing. Here, we investigate the fingering dynamics of shear-thinning viscoelastic droplets impacting superhydrophobic surfaces. At low polymer concentrations, liquid elasticity promotes the emergence of elongated fingers while simultaneously stabilizing them against breakup, thereby suppressing splashing. In contrast, an increasing polymer concentration enhances viscous damping, reducing the number of fingers and ultimately suppressing the fingering instability. Our results indicate that the onset of fingering is governed by the interplay of inertia, surface tension, and viscous stresses, while the number of fingers scales robustly with the Weber number. This highlights the dominance of inertia–capillary dynamics in our range of Weber numbers once the instability is triggered. Remarkably, all impact outcomes resulted in complete rebound, in contrast to a previous observation for viscoelastic droplets. Finally, we employ a theoretical framework to predict the temporal evolution of the mean ligament length across polymer concentrations, providing quantitative insight into how elasticity modifies drop retraction dynamics.

## Graphical abstract

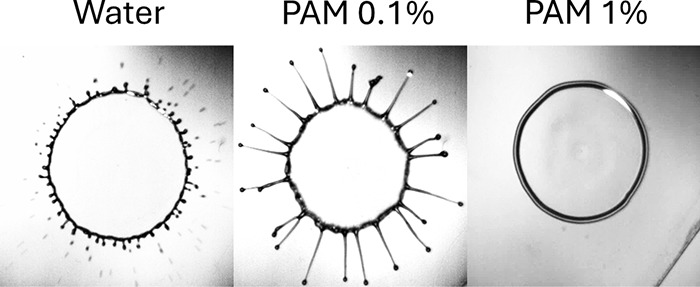

## Introduction

Drop impact is a phenomenon with significant relevance in a variety of applications such as inkjet printing,^1,2^ self-cleaning,^3–6^ and other surface coating processes.^7,8^ It is known that the outcome of drop impact can be controlled by the surface and liquid properties.^9^ These outcomes can be divided into deposition, rebound, and splashing behavior.^10^ The splashing occurs when drops hit a surface at high velocities, where a significant amount of impact energy leads to the formation of fingers during the drop spreading. The onset and growth of these fingers is named “fingering” instability, which is typically associated with Rayleigh–Taylor instability.^11–15^ In this case, a droplet (denser fluid) after impacting a solid surface gives rise to a spreading lamella that is decelerated in air (less dense fluid). This leads to perturbations that lead to the formation of fingers, which is similar to the principle of Rayleigh–Taylor instability.^16^ Furthermore, the fingers can subsequently break up into secondary jet drops through Rayleigh–Plateau instability. Suppressing such instabilities and associated splashing behavior is crucial for applications that rely on controlled droplet deposition, including inkjet printing^17,18^ and agricultural spraying.^19,20^ In the latter case, the target surfaces are often hydrophobic or superhydrophobic plant leaves, as their water-repellent nature hinders the complete deposition of small droplets.

Different parameters dependent on the liquid and surface properties have been identified as key factors governing the splashing dynamics, including wetting behavior,^21^ surface roughness,^22–24^ surface tension,^25^ viscosity,^21,26^ droplet charge and surface dielectric effects,^27^ droplet shape,^28^ and surface temperature.^29^ These properties are closely linked to the onset of fingering and the number of fingers formed during the spreading phase. In this context, the dimensionless Weber number (*We* = *ρD*_0_ *v*_0_^2^/σ) and Reynolds number (*Re* = *ρv*_0_ *D*_0_/η) (with ρ denoting the density of liquid, *v*_0_ the impact velocity, *D*_0_ the initial droplet diameter, σ the surface tension coefficient, and η the viscosity of liquid) are commonly employed to characterize the process, with various empirical and Rayleigh–Taylor-based scaling laws proposed to predict the number of fingers at the spreading rim. However, most of these studies have focused on Newtonian droplets, leaving the impact of non-Newtonian viscoelastic droplets largely unexplored, particularly in regimes where fingering instability and splashing occur. When the solid surface repels water, i.e., superhydrophobic, the viscoelasticity imparted by polymers in water has been proposed as a method to suppress droplet rebound,^30–33^ an important issue for applications with self-cleaning purposes. Both elongational viscosity and polymer-induced normal stresses have been identified as primary mechanisms opposing the receding motion of the impacting droplets. The dynamics becomes even more complex when the viscoelastic liquid is also shear-thinning, as its viscosity decreases with an increase in contact-line velocity. Recent studies have shown that the rebound dynamics of non-Newtonian droplets on superhydrophobic surfaces are more complex than previously assumed. Rebound suppression can depend sensitively on viscoelastic stresses, shear thinning, the interplay between dynamic and capillary pressures,^34^ and substrate stiffness.^35^ The elastic energy release during viscoelastic droplet retraction has even been linked to reduced contact times on superhydrophobic surfaces,^36^ without necessarily rebound suppression. Since these impact dynamics are governed by strong extensional stresses, they naturally connect to a broader class of viscoelastic phenomena in which fluid elements undergo rapid stretching. In this context, long-lived ligaments and delayed pinch-off, characteristic of viscoelastic flows, have also been reported in recent experimental and theoretical works,^37^ including observations of persistent filament formation in viscoelastic jets and droplets. Taken together, these findings highlight that the impact of the droplet on textured surfaces involves a coupled response governed by elasticity, wetting, and microstructure, making predictive characterization particularly challenging. Other recent studies have explored the impact dynamics of shear thickening droplets, evidencing a counterintuitive liquid-like response at the onset of impact^38^ followed by a solid-like transition as the shear rate decreases. This behavior further illustrates the diverse and complex ways in which non-Newtonian rheology influences droplet impact dynamics.

Here, we study the fingering formation of viscoelastic shear-thinning drops impacting a superhydrophobic surface at high impact speeds. We found that elongated and well-defined ligament-like fingers emerge from the spreading rim upon impact, without suppressing droplet rebound. The fingers can retract in unison with the receding contact line without undergoing splashing over a wide range of impact speeds, a behavior attributed to the elasticity of the liquid. Our experiments reveal that the onset of fingering is driven by inertia but resisted by capillary and viscous forces, which dampen the perturbations. In contrast, once the instability arises, the number of fingers is mainly dependent on the Weber number, as shear thinning reduces the effective viscosity and keeps it mostly unchanged at high shear rates. Finally, we propose a theoretical model in which ligament stretching is sustained by the combined pulling action of capillary forces and elastic stresses, providing a quantitative framework for retraction dynamics.

## Experimental Methods

### Sample Preparation

Microscopic glass slides (75 mm × 50 mm × 1 mm) were oxygen plasma activated (70 W, 5 min) to remove impurities. Later, the substrates were spray coated three times with silica nanoparticles suspended in isopropanol (Glaco Mirror Coat “Zero” from Soft99 Co.). The samples were left to dry at room temperature for 10 min. The spray coating process led to the formation of a silica nanoparticle layer (∼2 μm thick), providing sufficient roughness to render the surface superhydrophobic.

### Drop Impact Experiments

Microliter sized drops (diameter *D*_0_ = 2.5 mm) were dispensed onto a superhydrophobic surface coated with silanized silica nanoparticles from Glaco Soft99, at heights from 5 to 70 cm (impact speed *v*_0_ = 1.4–3.7 m/s). The impact velocity was calculated using the free-fall relation $v_{0}=\sqrt{2\mathrm{gH}}$ , where *H* is the release height (distance from nozzle to surface) and *g* is the gravitational acceleration (9.81 m/s^2^). The air drag force on the droplet was estimated from Stokes’s law *F*_D_ = 6*πη*_air_ *R*_0_ *v*_0_, where η_air_ is the air viscosity (18 μPa s) and *R*_0_ is the droplet radius. Within the range of release heights used, *F*_D_ reaches at most 1.7% of the droplet gravitational force *F*_g_ = *Vρg* = 8.5 μL, with *V* being the droplet volume, assuming a spherical shape. The impact was recorded by two high-speed cameras (Dantec dynamics, Denmark) at up to 7300 frames per second, for capturing the side and bottom views (Figure 1a). One mirror was placed surrounding the needle to deflect the light coming from a LED light source (KL 2500, Schott), while a second beneath the sample and inclined 45° to obtain a defined image of the drop bottom. The surface was placed on an aluminum sample holder at 0° of inclination. Experiments were repeated at least four times.

**Figure 1:**
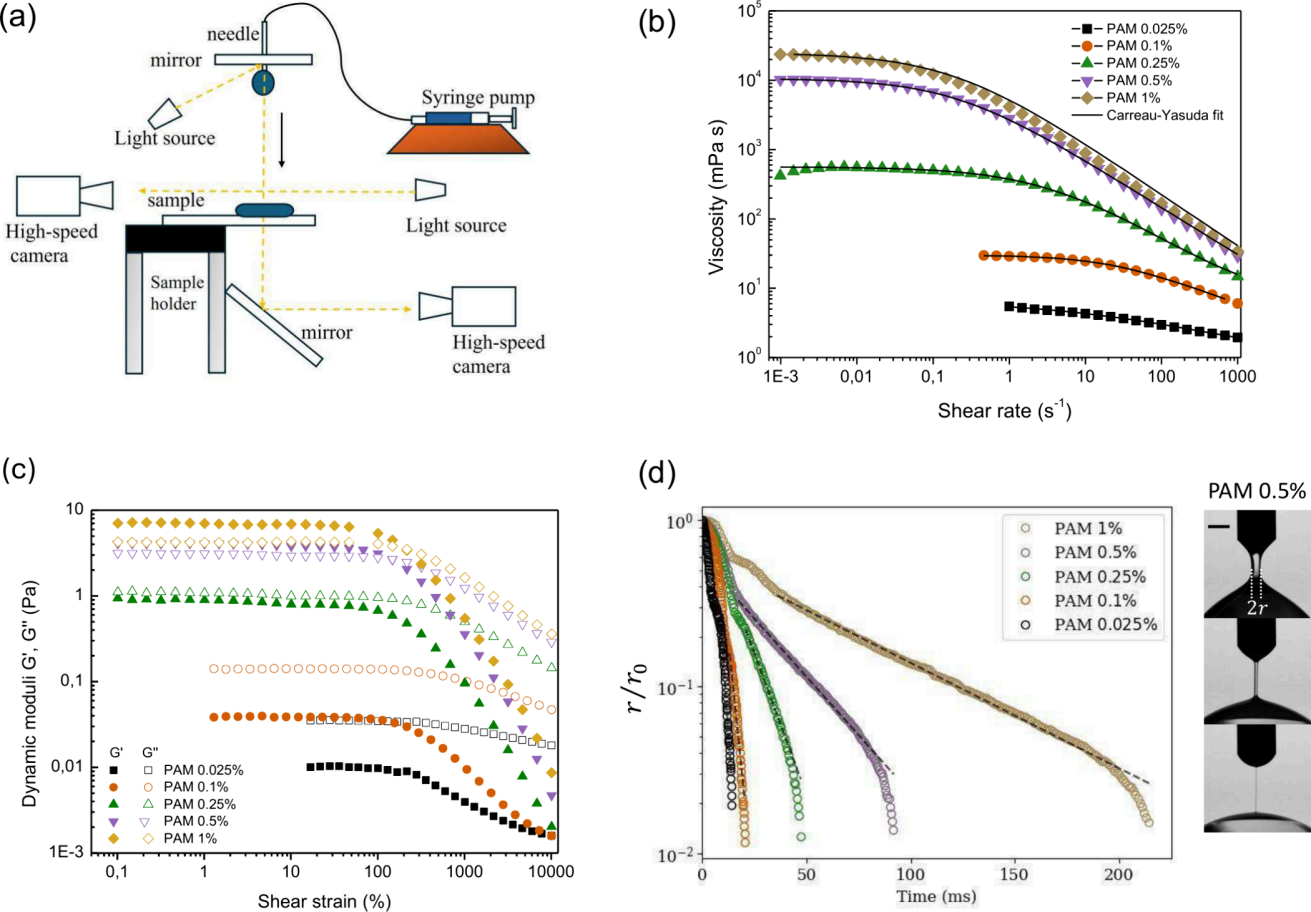
(a) Schematic of the experimental setup. (b) Viscosity as a function of shear rate for different PAM concentrations. (c) Dynamic moduli as a function of shear strain. (d) Evolution of minimum neck radius *r* over time from DoS experiments at different PAM concentrations. Dashed lines correspond to the fitting exp­(−*t*/3λ_ex_). The scale bar corresponds to 1 mm.

### Viscoelastic Solution Preparation and Rheometry Measurements

Viscoelastic solutions were prepared by mixing polyacrylamide (FLoPAM AM934SH, SNF, weight-average molecular weight *M*_w_ > 15 × 10^6^ Da) in deionized water (18 MΩ) for about 8 h at 1000 ± 50 rpm at concentrations of 250, 1000, 2500, 5000, and 10 000 ppm (denoted as PAM 0.025%, 0.1%, 0.25%, 0.5%, and 1%, respectively). In addition to the non-Newtonian solution, one glycerol solution (20% (v/v)) was prepared by mixing molecular biology-grade glycerol (Sigma-Aldrich) with deionized water. Our polymeric solutions do not retain a measurable volume fraction of entrained gas and therefore do not alter the bulk density appreciably. Glycerol mixtures have been reported to produce an only minor change in density in water (≈5%) in the concentration studied.^39^ Pendant droplet experiments evidenced differences of less than 1% and 4% for PAM 1% and 20% glycerol solutions, respectively (Figure S2 and section II of the Supporting Information). Consequently, for capillary and interfacial calculations, we assume the solutions have the same bulk density and surface tension as water: ρ = 1000 kg/m^3^ and σ = 72 mN/m.

Rheometry of the fluid samples is performed with an Anton Paar MCR 702e Space instrument (Anton Paar, Austria), using a 50 mm cone/plate measuring geometry set (1° cone angle, 93 μm truncation). Steady shear tests in shear rate range γ̇ ∈ [10^–3^, 10^3^] s^–1^ and oscillatory tests were performed, namely, strain amplitude sweep γ ∈ [10^–2^, 10^4^] and frequency sweep ω ∈ [10^–2^, 300] rad/s. The rheological measurements were performed at lab temperature, similar to the environment for the droplet impact experiments. All polymer concentrations show shear-thinning attributes (Figure 1b). The reciprocal of the frequency at the crossover point of the dynamic moduli (*G*′ and *G*″) in the frequency sweep test is taken as an estimation of elastic relaxation time scales λ_e_. Elastic modulus *G* was obtained by the plateau value of the *G*′ curves. The plot of dynamic moduli versus shear strain (Figure 1c) shows that as the PAM concentration increases to 0.25%, viscous properties dominate (*G*′/*G*″ < 1), while above that concentration, the elastic part becomes more dominant (*G*′/*G*″ > 1). The extent of shear thinning increases, and the zero-shear viscosity η_0_ plateau moves to lower shear rate ranges with an increase in the concentration of the polymer solutions. From the lowest to highest PAM concentration, zero shear viscosities range from ∼7 to 25 × 10^3^ mPa s.

### Contact Angle Measurements

For contact angle measurements, a goniometer drop shape analyzer (DSA25, Krüss) was used. Static contact angle were measured by depositing a 8 μL drop of Milli-Q water (18 MΩ) on the surface. The contact angle was measured by the tangent method fit of the device. All concentrations showed the same static contact angle θ_s_ of 165 ± 2° (see Figure S1), excluding any difference in the surface adhesion to the droplet dynamics for each case. To measure the advancing and receding contact angles, 5 μL was first deposited on the sample. Subsequently, the needle was adjusted to be in the middle of the drop. Afterward, the volume was increased to 10 μL at a flow rate of 0.5 μL/s. Then the drop was deflated at the same flow rate. All liquids showed a contact angle hysteresis of ∼5°

### Extensional Rheology

Drop onto surface capillary breakup rheology (DoS-CaBER) was performed to obtain the extensional relaxation time of polymers (λ_ex_). We generate a pendant droplet from a nozzle. A hydrophilic glass previously rinsed with ethanol was placed horizontally beneath the droplet and approached it gently. When the droplet and surface make contact with each other, the liquid spreads radially over the surface, creating a thinning capillary bridge. The process is recorded by a high-speed camera at up to 8900 fps. The Bond number of the experiment is *Bo* = *ρh*_0_ *l*_0_ *g*/σ ≈ 0.5,^40^ where *h*_0_ is the nozzle radius and *l*_0_ the distance from the nozzle to the substrate. The minimum thickness of the bridge is measured over time by MATLAB and fitted with an exponential function exp­(−*t*/3λ_ex_)^41,42^ (Figure 1d), where λ_ex_ is the extensional relaxation time of the non-Newtonian liquids. Contact angles between PAM droplets and the glass slide ranged from 8° to 16° (Figure S3 and section IV of the Supporting Information). Extensional relaxation time scales were between 0.6 and 22 ms for the range of polymer concentrations considered in this study. All relevant rheological parameters of the different fluids used η_0_, relaxation times λ_ext_ and λ, *G*, and infinite viscosity η_inf_, which are summarized in Table 1. η_inf_ was obtained by fitting the Carreau–Yasuda model^43^ to viscosity–shear rate curves (see section III of the Supporting Information).

**Table 1:** Rheological and Extensional Parameters for the Tested Solutions (zero-shear viscosity *η*_0_, infinite viscosity *η*_inf_, elastic modulus *G*, elastic relaxation time *λ*, and extensional relaxation time *λ*_ex_)

liquid	η_0_ (mPa s)	η_inf_ (mPa s)	*G* (Pa)	λ_ext_ (ms)	λ (s)
water	1	1	0	0	0
PAM 0.025%	6.95	1.16	0.007	0.64 ± 0.18	0.10
PAM 0.10%	29.93	0.12	0.05	1.18 ± 0.26	0.24
PAM 0.25%	563.3	6.36	0.94	4.26 ± 0.47	0.85
PAM 0.50%	10570	1.33	4.09	10.35 ± 0.92	2.86
PAM 1.00%	24810	1.50	7.06	21.83 ± 1.93	6.25
20% glycerol	2.6	2.6	0	0	0

## Results and Discussion

In this section, we will discuss the fingering instability induced by the droplet impact of viscoelastic droplets. The results are described in terms of the following dimensionless numbers: Weber number, Reynolds number, elastocapillary number, and Ohnesorge number, which can be obtained from Buckingham PI model derivation (section V of the Supporting Information).

### Effective Viscosity

To better describe the shear-thinning behavior of non-Newtonian droplets, we defined effective viscosity η_eff_ as the representative viscosity of the droplet impact process during spreading. We used the Carreau–Yasuda model^43^ and the corresponding fitting parameters (see section III of the Supporting Information) of our fluids to estimate η_eff_ (Figure 2a), considering a characteristic shear rate during droplet spreading as γ̇ = *v*_0_/*D*_0_, which is an expression commonly used in previous studies.^44–47^ Note that the initial viscosity of viscoelastic droplets prior to impact (equivalent to the zero-shear viscosity shown in Figure 1b) significantly decreases due to the high shear rates, in such a way that the increasing *We* slightly reduces η_eff_. Introducing the term η_eff_ will help us in the following sections compare droplet dynamics between the Newtonian and non-Newtonian scenario.

**Figure 2:**
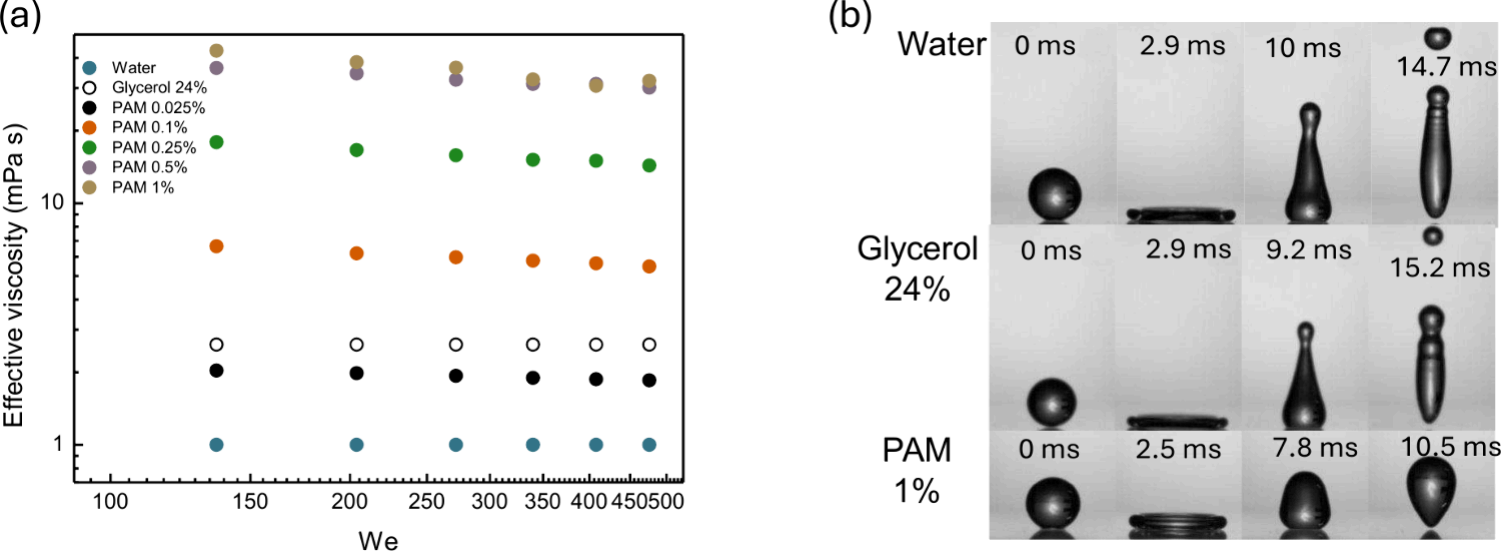
(a) Effective viscosity as a function of Weber number. Values were obtained from the Carreau–Yasuda model based on the characteristic shear rate during droplet impact. (b) Water droplet, 20% water/glycerol mixture, and PAM 1% impacting on a superhydrophobic surface coated with Glaco at *We* = 34.

### Fingering Formation

When a water droplet with *D*_0_ = 2.5 mm impacts a Glaco superhydrophobic surface when 1 ≤ *We* ≤ 136, a complete rebound takes place. We observed the same behavior for the viscoelastic cases and the water/glycerol (Figure 2b) mixture at 20% (2.6 mPa s). When *We* > 136, we classified the drop impact outcomes into three regimes (Figure 3): (1) fingering with splashing, (2) elongated fingering without splashing, and (3) no fingering. Regime 1 was observed for the Newtonian cases (water and 20% water/glycerol; 136 ≤ *We* ≤ 476). Fingers form continuously immediately upon impact as the lamella starts to grow, subsequently breaking up during the spreading phase (Figure 3a,b). Adding small amounts of polymer in water (0.025% and 0.1%) leads to regime 2, where elongated ligaments during the spreading and receding phases arise (Figure 3c and Figure S4). In particular, comparing WG 20% with PAM 0.025% , which have similar effective viscosities, it is clear that the difference in the fingering ligament elongation can be attributed to the elastic properties of the fluid imparted by PAM. This behavior of the elongated ligament resembles the sheet expansion of mucosalivary fluid, which is viscoelastic.^48^ Although weakly viscous droplets can also develop a few elongated fingers at very high impact speeds, these ligaments are unstable and eventually break up (Figure 3b). This highlights how elasticity stabilizes the rim, allowing a more symmetric ligament to grow while preventing breakup during both spreading and receding. Viscoelastic ligaments were observed for all of the ranges of *We* explored (136 ≤ *We* ≤ 476). Notably, splashing behavior did not occur for viscoelastic drops; therefore, ligaments retract and tend to merge with one neighbor until returning to the primary drop. Afterward, this drop rebounds completely from the surface, as reported in our previous work with the same liquid and surface^49^ (Figure S4). During the merging process, two nearby ligaments (or fingers) can coalesce into a single ligament, which may itself be subdivided into more fingers. As the polymer concentration increases (≥0.25%), we observe a suppression of the fingering instability, and the spreading droplet develops a toroidal-like morphology across the entire range of *We* (Figure 3d). To determine whether this behavior arises from viscous or elastic effects, we compared viscoelastic droplets with a 75% water/glycerol mixture under the same impact speed (Figure 3d,e). The water/glycerol mixture has a viscosity of 41 mPa s, of the same order of magnitude of the effective viscosities of PAM for concentrations above 0.25% (24–37 mPa s). Notably, the 75% glycerol droplet exhibits the same toroidal spreading shape (Figure S5). This comparison indicates that viscosity, rather than elasticity, is the dominant factor in suppressing fingering.

**Figure 3:**
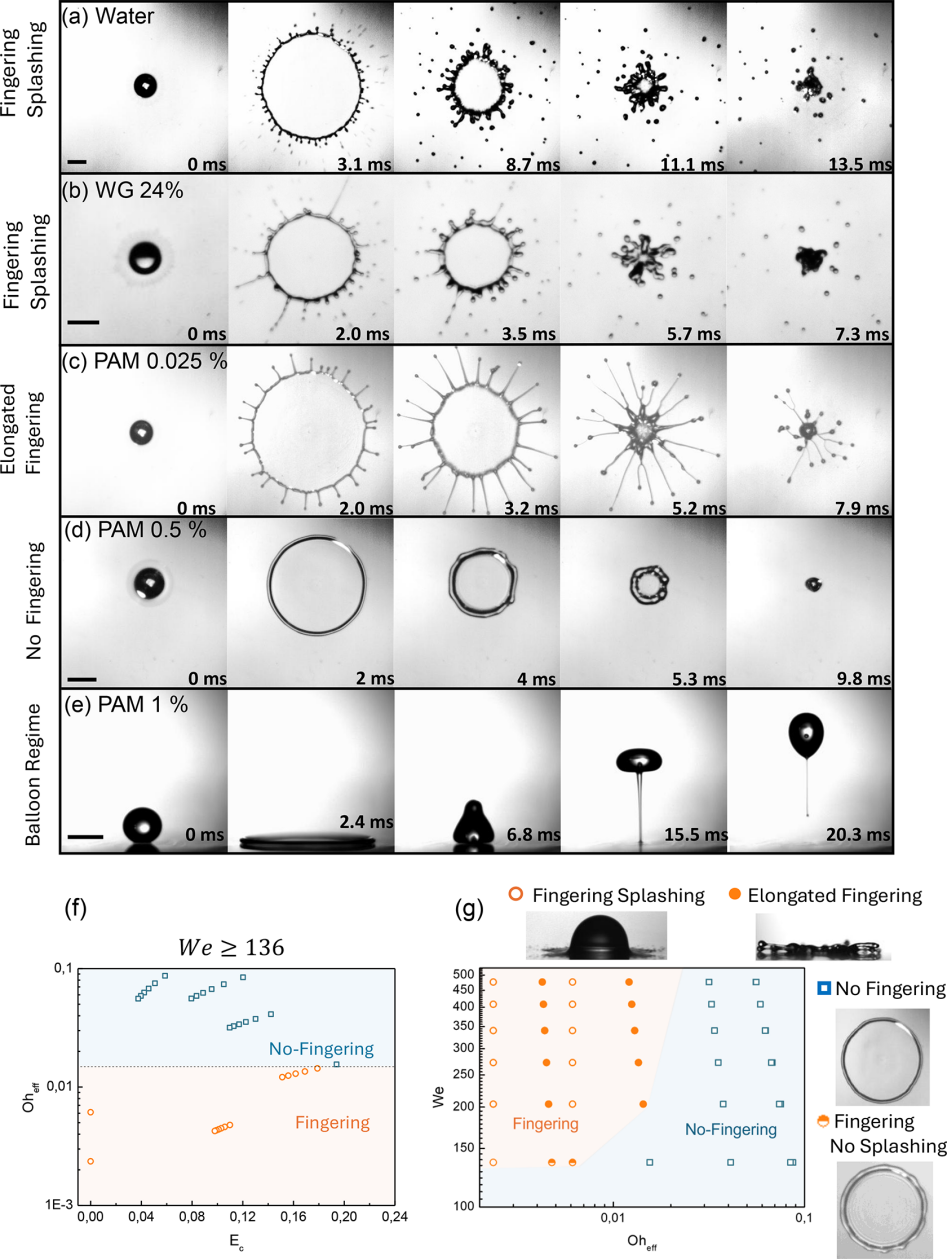
(a–e) Bottom view of a droplet impacting a superhydrophobic Glaco surface for water, a 20% water/glycerol solution (WG 20%), PAM 0.25% and 0.5% (all at *We* = 476), and PAM 1% (at *We* = 204). (f) Phase diagram in terms of *Oh*_eff_ and *E*_c_ for *We* > 136. (g) Phase diagram with different droplet impact outcomes in terms of *We* and *Oh*_eff_. Snapshots on the right represent the side and bottom views of different regimes identified. The scale bar represents 2.5 mm. Panels a, c, and d have the same magnification.

While the early time suppression of fingering is governed primarily by viscosity, the subsequent receding dynamics reveal behaviors unique to viscoelastic droplets. As the droplet recedes, a vertically growing ligament emerges from the contact line, decreasing in thickness until it detaches completely from the surface (Figure 3e and Figures S4 and S6). This behavior, identified in our previous work as the balloon regime,^49^ arises from the combined effects of liquid impalement (Cassie–Wenzel transition) into the surface microstructures and liquid elasticity. The penetration into the microstructure spacing increases the adhesion and reduces the contact-line velocity, which favors the vertical growth of ligaments until full detachment is achieved. Although the fingering scenario also shows such vertical ligaments due to impalement, their growth is less pronounced with a faster-thinning behavior (Figure S4).

### Threshold for Fingering Instability

The suppression of the splashing behavior and fingering by increasing polymer concentration suggests that these outcomes are strongly affected by the additional energy dissipation imparted by viscosity and elasticity. Consequently, at a higher polymer concentration, a greater impact energy should be required to trigger fingering instability. To disentangle whether viscosity or elasticity primarily governs the emergence of fingers, we represented the outcomes of droplet impact in two phase diagrams constructed from three dimensionless numbers: the Weber number, the effective Ohnesorge number ${Oh}_{\mathrm{eff}}=\eta_{\mathrm{eff}}/\sqrt{\rho \sigma D_{0}}$ (ratio of viscous to inertial and surface tension forces), and the elastocapillary number *E*_c_ = *G*_eff_ *D*_0_/σ (elastic to capillary stresses), where we introduce the effective elastic modulus *G*_eff_ ≈ η_eff_/λ_ex_ for non-Newtonian liquids. Note that *G*_eff_ is a shear-dependent quantity and is not the linear elastic modulus of the polymer model; it is a constructed parameter used to characterize the elastic contribution during impact. Therefore, for Newtonian droplets, no elastic relaxation exists and the definition of *G*_eff_ does not apply. Therefore, in this case, we set *G*_eff_ = 0 by convention, which yields *E*_c_ = 0 in the absence of elasticity. It is important to emphasize that λ_ex_ was used in the definition of *G*_eff_ because it more accurately reflects the extensional-flow character of the viscoelastic fingering instability.

We defined the high Weber number regime for *We* ≥ 136, where fingering instability starts to occur in some cases. Plotting *Oh*_eff_ as a function of *E*_c_ (Figure 3f) clearly separates cases with and without fingering. Fingering occurs at low *Oh*_eff_ values (orange region), while no fingering is observed at high *Oh*_eff_ values (blue region). In contrast, the data are broadly distributed across the entire *E*_c_ range, without a clear trend. This indicates that, within the explored parameter space, viscous forces dominate the onset of fingering, whereas elasticity plays only a secondary role. When viscosity is sufficiently low, inertia can destabilize the rim and promote finger formation. Therefore, inertia can then be considered as the driving force at the spreading rim, while resistive forces arise mainly from viscosity and surface tension.

Now that the dominant forces are distinguished, we can represent the impact outcomes in terms of *We* and *Oh*_eff_ (Figure 3g). The threshold (or critical) *We* to trigger fingering instability increases as *Oh*_eff_ decreases. This indicates that greater energy dissipation, here mainly viscous, will require greater inertia at the rim to overcome damping and promote finger formation. Notably, both the 20% water/glycerol mixture and the 0.025% PAM solution, which have similar viscosities (Figure 2a), exhibit the same *We* threshold for fingering instability without breakup. For water, the same threshold is shown in the plot; however, this does not correspond to the actual onset of protrusions since our experiments did not probe the precise *We* at which water forms fingers without splashing. In contrast, for glycerol and PAM 0.025%, no splashing was observed at the assigned threshold, only protrusions, indicating that the true onset *We* must be close to the reported value. Determining the exact threshold for every case would require prohibitively fine experimental resolution, but the present approximation is sufficient to capture the relevant physical trends.

### Suppression of Antirebound Effects

We observe complete rebound for all polymer concentrations across the entire range of the explored *We* (34 ≤ *We* ≤ 476). In the low-*We* regime (*We* ≤ 136), this behavior is consistent with three complementary effects. First, the substrates used here exhibit extremely low contact angle hysteresis (θ_s_ ≈ 165°; *Δθ* ≤ 5°), which minimizes contact-line pinning and the associated energy loss. In fact, prior computational work has shown that when the wetting properties remain unchanged, antirebound effects do not occur for viscoelastic droplets.^50^ Second, the shear-thinning behavior of polymer solutions significantly decreases their zero-shear viscosity during the high-shear rate spreading stage, reducing viscous dissipation and leaving more mechanical energy available for rebound. Third, a portion of the droplet kinetic energy is stored elastically as polymers stretch during spreading and then released during retraction, potentially accelerating contact-line motion.^36^ All of these effects explain why highly viscous droplets still rebound in our experiments. We emphasize that this explanation addresses the low-*We* regime only; the physics for *We* > 136 (local Cassie–Wenzel transition and vertical ligament detachment) have been attributed to elastic forces exceeding adhesion at the impaled region, which is discussed in our previous work.^49^

### Spreading Dynamics

The spreading dynamics was investigated to gain further insight into the dominant forces influencing the fingering instability. The maximum spreading factor β_max_ = *R*_max_/*R*_0_, where *R*_max_ is the maximum spreading radius, was then measured for all of the concentrations as a function of a modified impact parameter *P* = *WeRe*_eff_^–2/5^, where *Re*_eff_ is the effective Reynolds number *Re*_eff_ = *ρv*_0_ *D*_0_/η_eff_ (inertial to viscous forces). This parameter has been commonly used to describe the influence of viscous, inertial, and surface tension forces on droplet spreading.^51–53^ In our case, *P* also involves shear-thinning effects by considering *Re*_eff_. *R*_max_ was measured as average radius (*R*_*x*_ + *R*_*y*_)/2, obtained by manually placing an ellipse around the droplet footprint in the bottom-view high-speed images using ImageJ. Here, *R*_*x*_ and *R*_*y*_ correspond to the horizontal and vertical radii, respectively, of the spreading droplet (see the snapshots in Figure 4a).

**Figure 4:**
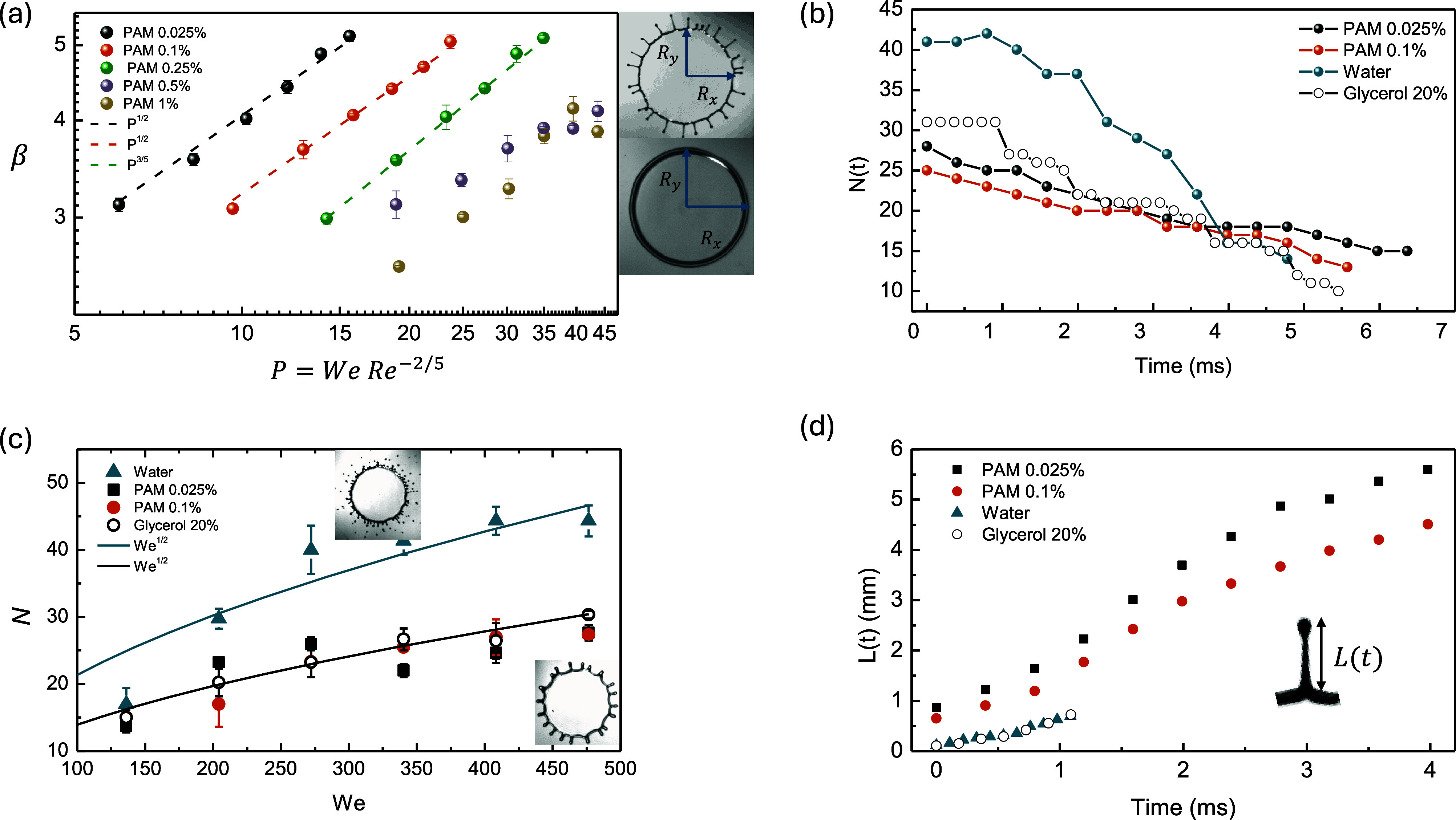
(a) Spreading parameter β = *R*_max_/*R*_0_ as a function of impact parameter *P* = *WeRe* ^–2/5^. (b) Evolution in time of the number of fingers for water, thr 20% water/glycerol mixture, PAM 0.025%, and PAM 0.1%. (c) Number of fingers at the maximum drop spreading as a function of *We*. (d) Ligament length over time for the Newtonian and non-Newtonian cases.

Our results indicate that β increases with *We* (Figure S7) and thus with *P* (Figure 4a). At a higher PAM concentration, β decreases due to the additional energy dissipation from viscoelasticity, which reduces the available energy to spread on the surface. For water, the spreading is primarily controlled by the competition between inertial and capillary forces, scaling with *We* as β_max_ ∼ *We* ^1/3^ (see Figure S7). Many studies have demonstrated numerically and experimentally that β_max_ ∼ *We* ^1/2^, but without taking splashing into account. When splashing occurs, volume loss contributes significantly to energy dissipation with a strong influence of viscous dissipation. This may reduce spreading and the power law coefficient. For the case of viscoelastic droplets, there is a clearer dependence on *P*. In particular, we found for the cases of fingering (0.025% and 0.1%) that β_max_ ∼ *P* ^1/2^, indicating that viscoelasticity can also influence spreading dynamics. Higher PAM concentrations, for which fingering does not occur, follow a different trend; for example, β_max_ ∼ *P* ^3/5^ for PAM 0.25%. Note that *R*_max_ for water and glycerol 20% is less than 0.025, and 0.1% due to the volume loss by splashing. Assuming a spheroidal shape for the ejected secondary drops and taking their average diameter, we calculated a volume loss during splashing of ∼1.5 μL. This volume is actually not negligible, significantly contributing to energy dissipation.

### Number of Fingers

We studied the dependence of the number of fingers on the different concentrations of PAM and *We* over time. For both Newtonian and non-Newtonian cases, the number of fingers *N*(*t*) decreased over time (Figure 4b) due to the merging of fingers until droplet rebound. This is a result of surface energy minimization. Water droplets exhibited a faster decrease in *N*(*t*) for water in comparison with glycerol mixtures and viscoelastic droplets. The variation of the wavelength over time can be estimated as λ­(*t*) ∼ 2*πR*(*t*)/*N*(*t*), where *R*(*t*) is the spreading rim radius over time. The splashing of water droplets leads to volume loss and a smaller *R*(*t*), which combined with a larger value of *N*(*t*) can favor the merging of fingers, increasing d*N*(*t*)/d*t*.

To elucidate the role of inertia, we measured the number of fingers, *N*, when the droplet reaches *R*_max_ as a function of *We*. We found that *N* increases with *We* for both Newtonian and non-Newtonian cases (Figure 4c). However, an increase in PAM concentration leads to a decrease in *N*. In fact, water droplets for *We* > 250 exhibited around 55% more fingers than viscoelastic drops. Interestingly, the non-Newtonian cases of 0.025% and 0.1% of PAM showed values of *N* quite close to those of the glycerol case. Moreover, the high effective shear rates (γ̇ > 792 s^–1^) at the contact line for the viscoelastic cases predict an effective viscosity (Carreau–Yasuda model) that barely decreases with *We*. Consequently, given the absence of elasticity for the water/glycerol mixture, our results suggest two important aspects. First, the droplet elasticity does not determine *N* but only delays finger growth and prevents its subsequent breakup. Second, increasing the viscosity decreases *N*. At this point, the shear-thinning behavior leads to roughly an unchanged viscosity in the *We* explored; thereby, the increase of *N* in our experiments is in fact strongly correlated to the increase in the impact energy (*We*).

Considering the Rayleigh–Taylor instability as the underlying mechanism of fingering, the spreading rim should experience a deceleration of *a* ∼ *v*_0_^2^/*D*_0_.^14,15^ According to Dai et al.^15^ and based on Rayleigh–Taylor instability, when *R* = *Re*/*We* ^3/4^ ≥ 10, *N* scales with *We* ^1/2^. This scaling is in reasonably good agreement with our experiments, taking into account our *R*_eff_ values, where *R* is greater than 10. Several studies have reported the same scaling law in the same regime, where the number of fingers appears to be insensitive to *Re*.^11,12,14,15^ Therefore, shear-thinning effects help to reduce the overall viscous damping, with the inertia as the driving force to trigger fingering.

### Ligament Dynamics

The emergence of fingers occurs due to the deceleration experienced by the rim due to Rayleigh–Taylor instability, which is on the order of *a* ∼ *v*_0_^2^/*D*_0_. For viscoelastic droplets, these fingers elongate in the form of ligaments, growing in time and suppressing the Rayleigh–Plateau instability. As the droplet reaches *R*_max_, the contact line stops spreading and the rim velocity is zero, but the fingers continue growing since there is still fluid coming from the bulk. At this point, the rim starts to recede, and the competition between capillary forces at the rim and inertial forces pulling fingers in the opposite direction increases the stretching of the ligaments. When the capillary force overcomes the inertial forces, ligaments are pulled back to the bulk until the droplet rebound without breaking up. This means that elastic forces are strong enough to resist ligament stretching.

The evolution of ligament length over time was measured for the Newtonian and non-Newtonian cases (Figure 4d) during the receding phase at *We* = 476. Maximum ligament length *L*_max_ for Newtonian droplets is reached just right before breakup, which can occur up to 1 ms after finger emergence. In contrast, ligaments of non-Newtonian droplets can stretch further due to fluid elasticity, reaching a maximum length of even 6 times larger than that in the Newtonian scenario. Although few fingers of 20% glycerol can grow similarly to non-Newtonian case, they are unstable and quickly break up during spreading (Figure 2b). Interestingly, we found that the growth velocity of ligaments, $\frac{dL\left(t\right)}{dt}$ , is quite close to the retraction velocity of the rim, $\frac{dR\left(t\right)}{dt}$ . This means that capillary forces at this point dominate the stretching of ligaments since they exceed the inertial forces acting on fingers moving opposite to the contact line. Furthermore, we found that the spreading parameter over time β­(*t*) collapses onto a single curve at the receding phase for the water, 0.025%, and 0.1% cases until a time lapse where fingers stop growing (Figure S8). Therefore, the retraction rate of these cases is the same: *V*_ret_^w^/*R*_max_ = *V*_ret_^*^/*R*_max_^*^, where *V*_ret_^w^ and *R*_max_ are the retraction velocity and maximum spreading radius of water, respectively, while the asterisk denotes the non-Newtonian case. Since the average growth velocity of ligaments *V*_l_ ≈ *V*_ret_^*^, we can express *L*_max_ as

1 $$L_{\max}\approx \frac{R_{\max}^{*}V_{\mathrm{ret}}\tau_{l}}{R_{\max}}$$

where τ_l_ is the retraction time of ligaments. Considering that *R*_max_^*^ ∼ *R*_0_ *P* ^1/2^, *R*_max_ ∼ *R*_0_ *We* ^1/3^, and *V*_ret_ = *V*_TC_, where *V*_TC_ = (σ/*ρh*)^1/2^ is the Taylor–Cullick velocity (*h* is the thickness of the lamella at the maximum spreading), and that τ_l_ is proportional to the inertio-capillary time scale τ_c_ = (*ρD*_0_^3^/σ)^1/2^, eq 1 yields

2 $$L_{\max}∼\frac{P^{1/2}V_{\mathrm{TC}}\tau_{c}}{We^{1/3}}$$

Equation 2serves to represent the magnitude of *L*_max_ when the suppression of splashing is guaranteed by the liquid elasticity, which can sustain the pulling stresses over the ligament, resulting from the interplay between capillary and inertial forces.

### Theoretical Model: Ligament Dynamics

Following the analysis of maximum finger length, we now develop a theoretical model for the temporal evolution of mean ligament length *L*(*t*). The present approach extends the rim-driven ligament growth framework of Wang and Bourouiba^54^ by incorporating polymeric stresses into the momentum balance. Critically, these extra stresses modulate rim deceleration, leading to an increase in the observed mean ligament growth as the elastic modulus of the viscoelastic liquid decreases.

In this formulation, ligament growth is analyzed by tracking the evolution of a single representative finger under the assumption that the ligament remains axisymmetric and cylindrical throughout its development. A local coordinate system is adopted, with *r* denoting the radial direction across mean ligament width *w* and *z* aligned with the ligament’s longitudinal axis, perpendicular to the rim (see Figure 5b). The fluid velocity feeding the ligament during its growth is denoted by *v*_l_, and the length of the ligament at time *t* is indicated by *L*(*t*). Ligament thickness *w* varies along the length of the ligament (see Figure 5a); however, in the considered theoretical model, we assume a uniform ligament width varying with time as $\mathrm{w̅}\left(t\right)=\sqrt{4\overset{®}{A_{\mathrm{lig}}\;}\left(t\right)/\pi }$ , where $\overset{®}{A_{\mathrm{lig}}\;}\left(t\right)$ corresponds to the average cross-sectional area of the ligament given by $\overset{®}{A_{\mathrm{lig}}\;}\left(t\right)=\left(1/L\left(t\right)\right)\int_{0}^{L\left(t\right)}A_{\mathrm{lig}}\left(z,t\right)\;dz$ where *A*_lig_(*z*, *t*) = *πw* ^2^(*z*, *t*)/4, indicating the local instantaneous cross-sectional area of the ligament at height *z*.

**Figure 5:**
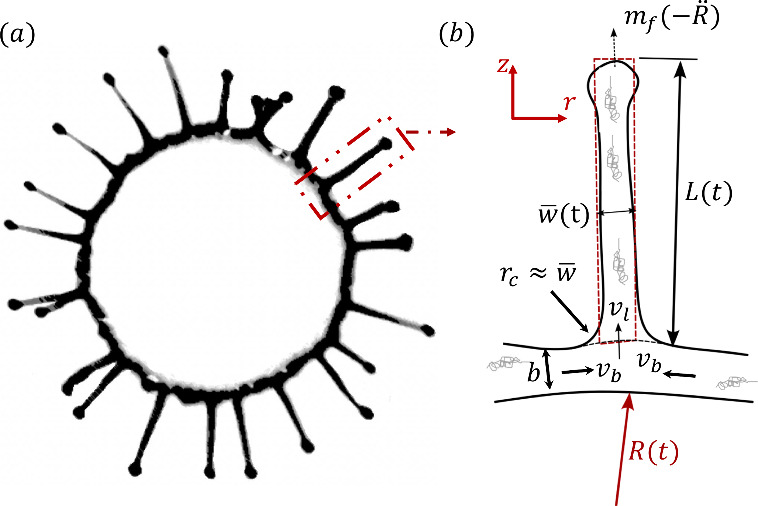
(a) Bottom view of a PAM 0.025% droplet impacting at *We* = 476 at *t* = 2.7 ms. (b) Schematic of the theoretical ligament-growth model, showing finger length *L*, mean width *w* , rim thickness *b*, inflow velocity into the ligament *v*_l_, rim-to-root velocity *v*_b_, local radius of curvature *r*_c_, rim radius *R*, and ligament deceleration force *m*_f_(−*R̈*).

A control volume is defined to encompass the entire ligament. Considering the uniform width approximation of the ligament, the mass conservation equation is then applied to this volume, relating the inflow rate to ligament growth as

3 $$\frac{dm_{f}}{dt}=\frac{d}{dt}\int_{0}^{L\left(t\right)}\rho A_{\mathrm{lig}}\left(z,t\right)\;dz=\rho \frac{\pi }{4}\frac{d}{dt}\left[{\mathrm{w̅}}^{2}\left(t\right)L\left(t\right)\right]=\rho \frac{\pi }{4}{\mathrm{w̅}}^{2}v_{l}$$

4 $$\frac{dL}{dt}+\frac{2L}{\mathrm{w̅}}\frac{d\mathrm{w̅}}{dt}=v_{l}$$

The momentum balance in the ligament is given as

5 $$\frac{d}{dt}\int_{0}^{L\left(t\right)}\rho A_{\mathrm{lig}}\left(z,t\right)u\left(z,t\right)\;dz=\rho \frac{\pi }{4}{\mathrm{w̅}}^{2}{v_{l}}^{2}-\pi \mathrm{w̅}\sigma +p\frac{\pi {\mathrm{w̅}}^{2}}{4}+m_{f}\left(-\mathrm{R̈}\right)-\tau_{p,\mathrm{zz}}\frac{\pi }{4}{\mathrm{w̅}}^{2}$$

where *u*(*z*, *t*) is the fluid velocity profile in the ligament and the first term on the right is the momentum influx entering the ligament. The second and third terms on the right correspond to the surface tension force and pressure force acting on the root of the ligament , respectively. In addition, *m*_f_(−*R̈*) = *ρπ* *w* ^2^ *LR̈*/4 is the fictitious body force due to the non-Galilean reference frame of the rim.^55^ The last term in the equation corresponds to the axial polymeric stress force at the base of the ligament.

If influx velocity *v*_l_ is known (see eq 4), then axial fluid velocity profile *u*(*z*, *t*) can be obtained from the continuity equation as

6 $$\frac{\partial }{\partial t}A_{\mathrm{lig}}\left(z,t\right)+\frac{\partial }{\partial z}\left[A_{\mathrm{lig}}\left(z,t\right)u\left(z,t\right)\right]=0$$

Under the uniform ligament width approximation, this simplifies to

7 $$\frac{d\mathrm{w̅}}{dt}+\frac{\mathrm{w̅}}{2}\frac{\partial u}{\partial z}=0$$

From eqs 4 and 7, we can obtain the fluid velocity profile as

8 $$\frac{\partial u}{\partial z}=-\frac{2}{\mathrm{w̅}}\frac{d\mathrm{w̅}}{dt}=\frac{1}{L}\left(\frac{dL}{dt}-v_{l}\right)\Rightarrow u\left(z,t\right)=\frac{z}{L}\left(\frac{dL}{dt}-v_{l}\right)+u\left(0,t\right)$$

where *u*(0, *t*) is the fluid velocity at the root of the ligament and is equal to the velocity of the fluid entering the ligament (*v*_l_).

The momentum balance equation (eq 5) can be rewritten using eqs 7 and 8 as

9 $$L\frac{d}{dt}\left(\frac{dL}{dt}+v_{l}\right)=v_{l}\left(v_{l}-\frac{dL}{dt}\right)-\frac{8\sigma }{\rho \mathrm{w̅}}+\frac{2p}{\rho }+2L\left(-\mathrm{R̈}\right)-\frac{2\tau_{p,\mathrm{zz}}}{\rho }$$

The curvature-induced pressure at the ligament root scales inversely with the local radius of curvature, *r*_c_, which can be approximated to the mean width of the ligament (see Figure 4b). Hence, substituting *p* = 2σ/ *w* into eq 9, we obtain

10 $$L\frac{d}{dt}\left(\frac{dL}{dt}+v_{l}\right)=v_{l}\left(v_{l}-\frac{dL}{dt}\right)-\frac{4\sigma }{\rho \mathrm{w̅}}+2L\left(-\mathrm{R̈}\right)-\frac{2\tau_{p,\mathrm{zz}}}{\rho }$$

Given τ_p,*zz*_, *v*_l_, and *R̈*, eqs 4 and 10 can be integrated to obtain the temporal evolution of mean ligament length *L*(*t*), provided that initial values of *L* and *w* are specified at the onset of rim deceleration (*t*_0_). In the experiments, the onset of rim deceleration *t*_0_ ≈ 2 ms from the time PAM 0.025% droplet hits the substrate and for PAM 1% droplet impacting at *We* = 476, *t*_0_ ≈ 2.7 ms.

To determine the polymeric stress component along the length of ligament τ_p,*zz*_, we solve for the evolution equation of conformation tensor *A*_*ij*_, which represents the average polymer deformation at the continuum scale. Assuming negligible polymeric stress at *t*_0_ (a strong assumption, as elastic stresses may persist from rim stretching), we solve for the uniaxial extensional equation

11 $$\frac{dA_{zz}}{dt}=2\mathrm{\varepsilon ̇}A_{zz}-\frac{f\left(A\right)}{\lambda_{\mathrm{ext}}}\left(A_{zz}-1\right),\mathrm{where}\;A_{zz}\left(t_{0}\right)=1$$

where λ_ext_ is the extensional relaxation time of polymer stress (0.6 ms for PAM 0.025% and 1 ms for PAM 0.1%). Strain rate *ε̇* is defined as

12 $$\mathrm{\varepsilon ̇}=\frac{1}{L}\frac{dL}{dt}$$

Stress relaxation function *f*(**A**) is dependent on the chosen constitutive relationship for simulating polymer dynamics. In this study, we employ the linear–Phan–Thien–Tanner (L-PTT) model, which enables the tuning of extensibility parameter α to capture the interplay between shear-thinning and extensional hardening behavior in viscoelastic materials,^56^ which is relevant for capturing ligament dynamics.^49^ The relaxation function for the L-PTT model^57^ is

13 f(A)=1+αtr(A−I)(A−I)

with α set to 0.01 for PAM 0.025% and 0.025 for PAM 0.1%, which are obtained as fitting parameters from the rheological measurements of steady-shear characteristic curves (Figure S9 and section VIII of the Supporting Information for details). The governing equations for the considered rheological model (L-PTT) are solved for the Couette flow configuration at different shear rates.

Polymeric stress component τ_p,*zz*_ is retrieved from the conformation tensor as

14 $$\tau_{p,\mathrm{zz}}=G_{\mathrm{eff}}\left(A_{zz}-1\right)$$

where *G*_eff_ is the effective elastic modulus of the viscoelastic material (*G*_eff_ = 3 Pa for PAM 0.025%, and *G*_eff_ = 5 Pa for PAM 0.1%). Note that *G*_eff_ incorporates shear thinning into ligament growth because μ_eff_ is taken from a Carreau–Yasuda fit evaluated at the characteristic impact shear rate.

To determine *v*_l_ for solving the system of eqs 4 and 10, we follow the same physical argument^54^ used to estimate the fluid speed entering the ligament from the rim–ligament junction. First, velocity *v*_b_ from the rim to the ligament junction is estimated using Bernoulli’s principle as $v_{b}=\sqrt{2\sigma /\rho r_{c}}$ . The curvature-induced pressure in the ligament (2σ/ *w* ) opposes the inflow (from the rim to the ligament), and thereby, rim deceleration *R̈* provides a driving force against this curvature-induced resistance. Specifically, in the rim, the curvature-induced pressure is 2σ/*b* (with *b* denoting the rim thickness). The force potential includes the rim deceleration term −ρ­(*r*_c_ + *b*/2)­(−*R̈*). Applying Bernoulli’s principle between the rim and ligament cross sections gives an expression for inflow velocity as

15 $$v_{l}=\sqrt{\left(2r_{c}+b\right)\left(-\mathrm{R̈}\right)+\frac{4\sigma }{\rho }\left(\frac{1}{b}-\frac{1}{\mathrm{w̅}}\right)}$$

Following the local instantaneous Bond number criterion *Bo* = *ρb* ^2^(−*R̈*)/σ = 1 by Wang et al.,^58^ the rim’s deceleration *R̈* can be linked to thickness *b* as (−*R̈*) = σ/*ρb* ^2^ (which can be applied for a range of viscous and elastic properties of the fluid). In order to check the validity of the relationship between *b* and *R̈*, we plot the experimental data of rim thickness for PAM 0.025% and PAM 0.1% droplets impacting at *We* = 476 in Figure 6. The corresponding relationship of $b=1.1\sqrt{\sigma /\left(\rho \left(-\mathrm{R̈}\right)\right)}$ is also plotted in the figure, and it shows a reasonable agreement with the experimental data. Due to limited temporal resolution in experiments, rim deceleration *R̈* data are noisy, so only valid experimental data points are retained. In addition, an empirical model is considered for rim radius *R*(*t*), which is discussed in the next section.

**Figure 6:**
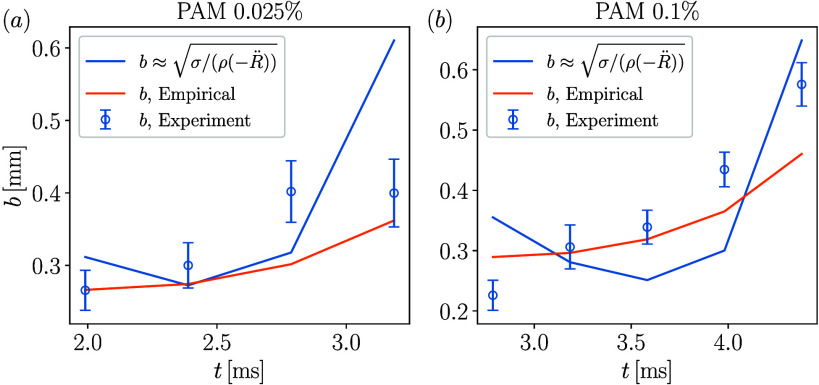
Temporal variation of rim thickness *b* for (a) PAM 0.025% and (b) PAM 0.1% droplets impacting at *We* = 476.

Influx velocity *v*_l_ can thus be written as

16 $$v_{l}=\sqrt{\frac{2\sigma }{\rho b}\left(\frac{1.21r_{c}}{b}+\frac{5.21}{2}-\frac{2b}{\mathrm{w̅}}\right)}$$

Integrating the closed system of eqs 4, 10, and 16 over time, with *L*(*t*_0_), *R*(*t*_0_), and *w* ­(*t*_0_) taken from experiments, yields temporal ligament length *L*(*t*). As shown in panels a and b of Figure 7, theoretical model predictions agree well with experimental measurements for PAM 0.025% and 0.1% at *We* = 476. The results confirm that the mean ligament model can capture viscoelastic ligament dynamics at high Weber numbers. In addition, the assumption of negligible polymeric stress at the onset of rim deceleration seems reasonable with the predicted mean elastic stresses of O(1) Pa for PAM 0.025% and O(10) Pa for PAM 0.1%. Locally along the ligament height, the polymeric stresses may be higher, counteracting capillary stresses and thereby preventing ligament breakup.

**Figure 7:**
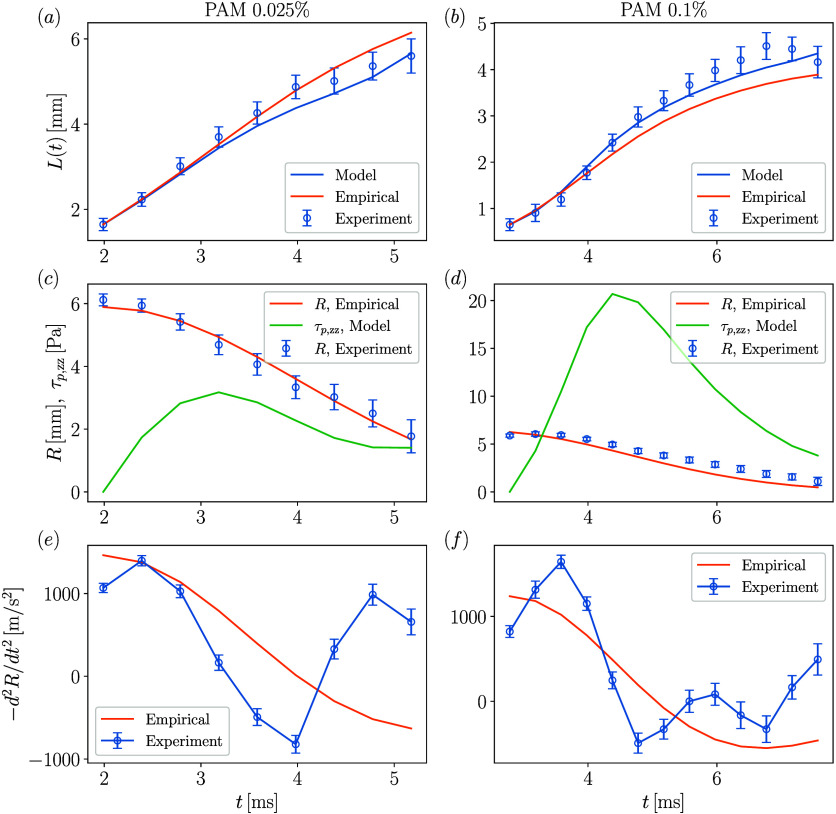
Temporal evolution of ligament length for (a) PAM 0.025% and (b) PAM 0.1% droplets impacting at *We* = 476. The corresponding rim radius *R* and polymeric stress evolution are shown in panels c and d, respectively. The respective rim decelerations are indicated in panels e and f.

### Empirical Modeling of Ligament Retraction

Having established the theoretical framework for predicting ligament growth, we now turn to the experimental observations of rim radius evolution. Since the temporal variation of *R*(*t*) plays a central role in driving ligament dynamics, an empirical model for the rim radius is developed to complement the theoretical approach and provide a direct link to measurable quantities.

Maximum rim radius *R*_max_^*^ can be predicted by the empirical relationship obtained from our previous spreading dynamics analysis:

17 $$R_{\max}^{*}∼R_{0}P^{1/2}$$

However, from the experimental *R*(*t*) data, we derive an empirical relationship for the temporal variation of rim radius *R* considering three different polymer concentrations (PAM 0.025%, 0.1%, and 0.25%) as

18 $$R\left(t\right)=R_{\max}^{*}\;\exp\left(-t^{2}/\left(0.06\sqrt{E_{c}}{\tau_{c}}^{2}\right)\right)$$

Panels c and d of Figure 7 compare the empirical fit with experiments, while panels e and f of Figure 7 show the rim deceleration for PAM 0.025% and 0.1%, respectively. Overall, the empirical fit performs well and can serve as an approximate predictive tool for mean ligament length evolution. The radius variation as obtained empirically in eq 18 describes a Gaussian decay, where the radius of the ligament decays from a maximum and symmetrically decreases over time. The term in the exponent ${\mathrm{t̂}}^{2}=t^{2}/\left(0.06\sqrt{E_{c}}{\tau_{c}}^{2}\right)$ is a squared nondimensional time, where *E*_c_^1/4^τ_c_ corresponds to the elasticity modification to inertio-capillary time scale τ_c_. Nondimensional time *t̂* quantifies how observed time *t* compares to the effective elasto-inertio-capillary time scale. When *t̂* ≪ 1, the exponent is near zero, so *R* ≈ *kR*_max_^*^, indicating that the initial plateau driven by inertia is resisting capillary collapse (*k* is a constant). When *t̂* ≫ 1, the Gaussian decay dominates the set by capillarity overcoming inertia, modulated by elasticity through *E*_c_. The form of empirical relationship as provided in eq 18 suggests that the inertia and capillarity primarily govern radius evolution, with elasticity modifying the time scale. The quadratic time dependence inside the exponent suggests that the process accelerates with time, consistent with the increase in capillary pressure as the radius decreases. Note the weak dependence of modification to the capillary time scale induced by elasticity as *E*_c_^1/4^. This indicates that for larger *E*_c_ values the elastic stresses counteract capillary forces extending the elasto-inertio-capillary phase before capillary dominance in the deceleration process. However, from Figure 7d, a higher concentration of PAM (thereby a larger *E*_c_) can show momentarily greater deceleration and overall can have minimal differences with respect to the considered concentrations in the empirical fit. Hence, for a robust understanding of the variation of rim radius with respect to polymer concentration, a data set with different concentrations and higher time resolution becomes necessary, which is not considered within the scope of the present work.

To summarize, we developed a theoretical framework to predict the temporal evolution of ligament length in high-Weber number impacts of viscoelastic (PAM–water) droplets on superhydrophobic surfaces. The model integrates mass and momentum conservation with polymeric stress evolution via the L-PTT constitutive equation, incorporating rim deceleration and curvature-induced pressure gradients as driving mechanisms for ligament elongation. The influx velocity from the rim into the ligament is derived from Bernoulli arguments, accounting for the interplay among rim curvature, ligament geometry, and polymer elasticity.

Model predictions show good agreement with high-speed imaging data for two PAM concentrations, capturing both the rate and the extent of ligament elongation. The results demonstrate that even small amounts of polymer significantly alter rim deceleration and elongation dynamics, leading to more persistent, elongated ligaments and suppression of splashing. The framework also highlights the role of mean polymeric stresses in opposing capillary-driven pinch-off, suggesting that local elastic stresses may be even larger and crucial to splash suppression.

## Conclusions

In this work, we investigated the fingering instability of viscoelastic droplets impacting superhydrophobic surfaces. Impact outcomes were classified as fingering or nonfingering in terms of effective Ohnesorge, Weber, and elastocapillary numbers. Our experiments show that the onset of fingering is governed by the competition among inertia, surface tension, and viscous stresses, with higher viscosity requiring larger Weber numbers to trigger fingering. At low polymer concentrations, fluid elasticity promotes finger elongation into ligaments, thereby suppressing splashing. In contrast to the rebound-suppressing behavior reported for viscoelastic droplets, all impacts across polymer concentrations resulted in complete rebound. We further found that the number of fingers scales robustly with the Weber number, consistent with Rayleigh–Taylor instability, as shear thinning reduces viscosity’s role as a dissipative mechanism. Ligament growth occurs at approximately the same rate as the receding droplet contact radius, evidencing the key role of capillary forces as the driving force of elongation. Finally, we proposed theoretical and empirical models to predict ligament length evolution, both in good agreement with experimental data. It should also be highlighted that there are several key assumptions such as axisymmetric and uniform width ligaments (thereby ignoring the thickness variations) in deriving the theoretical model for ligament length evolution. The polymeric stress is incorporated as a base-averaged term, and the prediction from the theoretical model depends on the influx velocity and the rim deceleration. The theoretical model can track the mean length of the ligament in its growth phase but does not account for end-pinching effects or secondary fragmentation. In addition, additional data at different concentrations can help in refining the empirical model. The above aspects can be considered as the scope of future work. Overall, the present findings provide new insight into droplet impact dynamics and applications involving droplet deposition, such as spray technologies, pathogen control, and chemical deposition.

## Supplementary Material



## Notes

Static contact angles of viscoelastic droplets, fitting parameters of the Carreau–Yasuda model, supporting figures for spreading and retraction dynamics, and additional experimental data (PDF)
