# Supplementary material for: Viscoelastic Fingering of Shear-Thinning Drops Impacting on Superhydrophobic Surfaces
Source: Langmuir. 2026 May 7;42(19):13925–37. doi: 10.1021/acs.langmuir.6c01487 (PMC13192325; doi:10.1021/acs.langmuir.6c01487)
Supplement: Supplementary file 1 [file la6c01487_si_001.pdf]

Supporting Information for: Viscoelastic fingering of shear-thinning drops impacting on superhydrophobic surfaces

Diego Díaz[1], Arivazhagan Geetha Balasubramanian[1,2], Kasra Amini[1], Shervin Bagheri[1] and Outi Tammisola[1,2]

[1] FLOW and Fluid Physics Laboratory, Dept. of Engineering Mechanics, KTH Royal Institute of Technology, 100 44 Stockholm, Sweden

[2] Swedish e-Science Research Centre, Dept. of Engineering Mechanics, KTH Royal Institute of Technology, 100 44 Stockholm, Sweden

Corresponding author email: didd@kth.se, shervin@mech.kth.se, outi@mech.kth.se

Number of pages: 10

Number of figures: 9

Number of tables: 2

Table of contents

S1. Static contact angle for different PAM concentrations.

S2. Pendant droplet experiment for water, glycerol 20% and PAM 1%.

S3. Snapshots of a droplet of PAM at 0.025% and 1% deposited on a glass slide, and static contact angle for different concentrations of PAM.

S4. Droplet of PAM 0.1% impacting a superhydrophobic surface at Weber number $We = 340$.

S5. Droplet of PAM 1% impacting a superhydrophobic surface and Water -glycerol droplet at 75% concentration impacting at the same speed the same surface.

S6. Droplet of 1% PAM impacting a superhydrophobic surface at Weber number $We = 272$.

S7. Spreading parameter as a function of Weber number.

S8. Spreading parameter as a function of time for water, PAM 0.025% and PAM 0.1%.

S9. The steady shear curves obtained for the Phan–Thien–Tanner model with PAM 0.025 % and PAM 0.1 %.

Table S1. Carreau-Yasuda fit parameters.

Table S2. Fitting parameters of Phan–Thien–Tanner model

I.- Static Contact angle

The static contact angle was measured by the sessile drop method, depositing a 8.5 µL drop on the superhydrophobic surface. All Polyacrylamide (PAM)-water mixtures showed the same contact angles, thereby the wetting properties are not affected by polymer concentration.

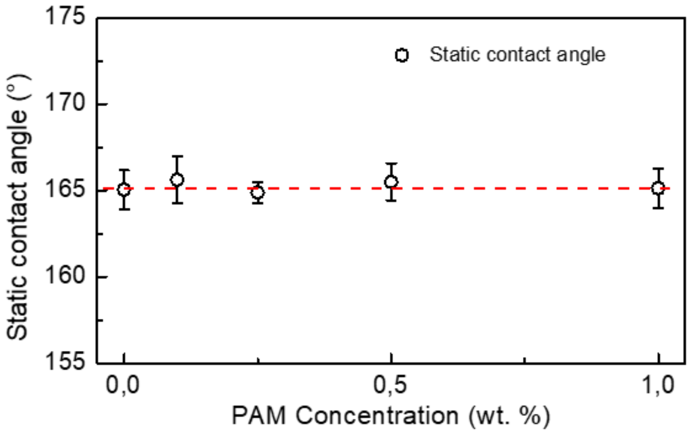

Fig S1. Static contact angle for different PAM concentrations. Red dashed line represents a guide to the eye.

II. Pendant droplet measurements

Pendant droplet measurements were performed on Goniometer device (DSA25, Krüss), by generating a droplet of ≈ 30 µL pending from a nozzle of 1.8 mm outer diameter. Surface tensions of PAM and glycerol droplets slightly change (Fig. S2).

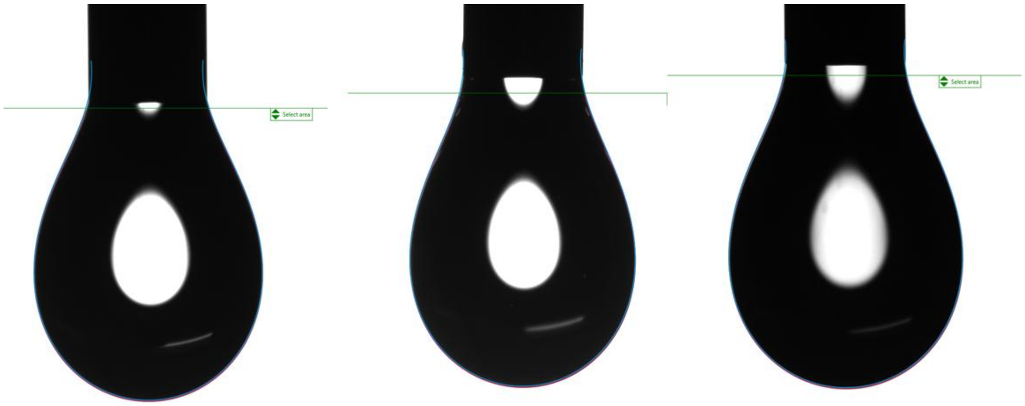

Water $\sigma = 71.92$ mN/m

Glycerol 20% $\sigma = 69.43$ mN/m

PAM 1% $\sigma = 71.54$ mN/m

Fig. S2. Pendant droplet experiment for water, glycerol 20% and PAM 1%.

III.- Carreau Yasuda model

The shear-thinning effects of the polymer solutions are described by the five-parameter Carreau-Yasuda model, characterized by the zero-shear viscosity η_0, the infinite-shear viscosity η_{inf}, the characteristic time λ_{CY} that defines the onset of shear-thinning, the power law index n, and the dimensionless Yasuda parameter a, describing the transition from the Newtonian plateau to the power-law region.

$$\eta(\dot{\gamma}) = \eta_{inf} + (\eta_0 - \eta_{inf})[1 + (\lambda_{CY}\dot{\gamma})^a]^{\frac{n-1}{a}} \quad (6)$$

Table S1. Carreau-Yasuda fit parameters.

Fluid Sample	η_0 [mPa.s]	η_{inf} [mPa.s]	λ [s]	n [-]	a [-]
PAM 1%	24810	1.505	7.436	0.2457	0.6509
PAM 0.5%	10570	1.333	4.783	0.3056	0.7001
PAM 0.25%	563.3	6.364	0.2409	0.2628	0.6181
PAM 0.1%	29.93	0.116	0.0461	0.5811	0.8753
PAM 0.025%	6.95	1.156	0.0006	0.00003	0.3084

IV. Contact angles of PAM on glass slides for DoS experiments

Contact angles of 6 µL drops of PAM in water at concentrations of 0.025, 0.1, 0.25 and 1% were measured using a Goniometer device (DSA25, Krüss) on glass slides previously rinsed with ethanol. Contact angles ranged between 6 to 16 degrees. A tangent fitting method provided by the software of the goniometer was used to determine the contact angle.

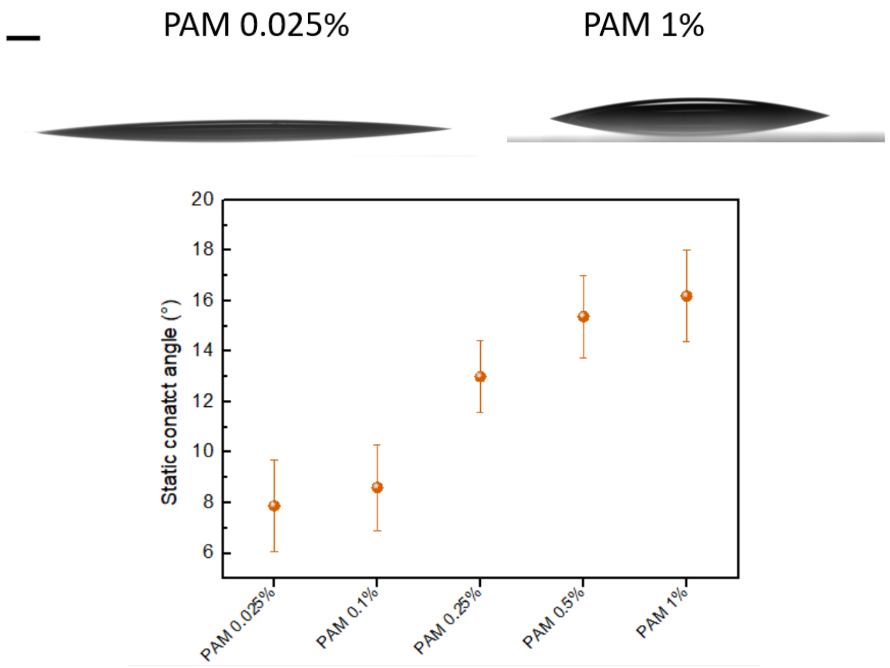

Fig. S3 Top: snapshots of a droplet of PAM at 0.025 and 1% deposited on a glass slide. Bottom: Static contact angle for different concentrations of PAM.

V. Buckingham PI model

In our experiments we have the following $n = 6$ six relevant parameters: $\rho, \sigma, v_0, D_0,\ \eta_{eff}$ and λ_e (liquid density, surface tension, impact speed, droplet diameter, effective viscosity and extensional relaxation time, respectively). Choosing $k = 3\ (M, L, T)$ independent variables: ρ, D_0 and v_0 we get $p = n - k = 3$ PI groups of the form

$$\Pi = \rho^a v_0^b D_0^c X$$

Where, X corresponds to the variables that are not independent. Here the dimensions can be expressed as: $[\rho] = ML^{-3}$, $[D_o] = L$, $[v_o] = LT^{-1}$, $[\eta_{eff}] = ML^{-1}T^{-1}$, $[\sigma] = LT^{-2}$ and $[\lambda_e] = L$

As a result, the first PI group is:

$$\Pi_1 = \rho^a v_0^b D_0^c\ \sigma$$

This leads to: $[\Pi_1] = M^{a+1}L^{-3a+b+c}T^{b-2}$, with $a = -1$, $b = -2$, $c = -1$ and then:

$$\Pi_1 = \rho^{-1} v_0^{-2} D_0^{-1}\ \sigma$$

Inverting we get the Weber number:

$$\Pi_1^{-1} = \frac{\rho D_0 v_0^2}{\sigma} = We$$

The second PI is $\Pi_2 = \rho^a v_0^b D_0^c\ \eta_{eff}$, leading to $[\Pi_1] = M^{a+1}L^{-3a+b+c}T^{b-1}$ and $a = -1$, $b = -1$, $c = -1$. Therefore:

$$\Pi_2 = \rho^{-1} v_0^{-1} D_0^{-1}\ \ \eta_{eff}$$

Inverting, we obtain the Reynolds number

$$\Pi_2^{-1} = \frac{\rho D_0 v_0}{\mu} = Re$$

Finally, the third PI group

$$\Pi_3 = \rho^a v_0^b D_0^c\ \lambda_e,$$

which leads to: $[\Pi_3] = M^a L^{-3a+b+c} T^{b+1}$, with $a = 0$, $b = 1$, $c = -1$ and then we obtain the Deborah number:

$$\Pi_3 = v_0 D_0^{-1} \lambda_e = De$$

Therefore, Π_2 and Π_3 justify the use of Ohnesorge number $Oh = \sqrt{We}/Re$ and Elastocapillary number$Ec = We/(ReDe)$ respectively obtained with inertia-capillary and capillary scaling, in the present study to describe the role of inertia, elasticity, viscosity and capillary effects in the fingering dynamics.

VI. Elongated spreading ligaments and Balloon regime

Low polymer concentrations (0.1% and 0.025% PAM) exhibited the formation of elongated fingers upon impact, which then retracts back together with the primary drop without breaking up (Fig. S4). During the receding phase, a ligament at the superhydrophobic surface arises, growing vertically until full detachment. Afterwards, this ligament retracts back to the primary drop. Increasing the polymer concentration suppresses the fingering instability (Fig. S5a) and enhances the emergence of a growing ligament from the surface (Fig. S6). Such behavior can be explained by the penetration of the liquid into the surface air pockets due to the high impact pressure.

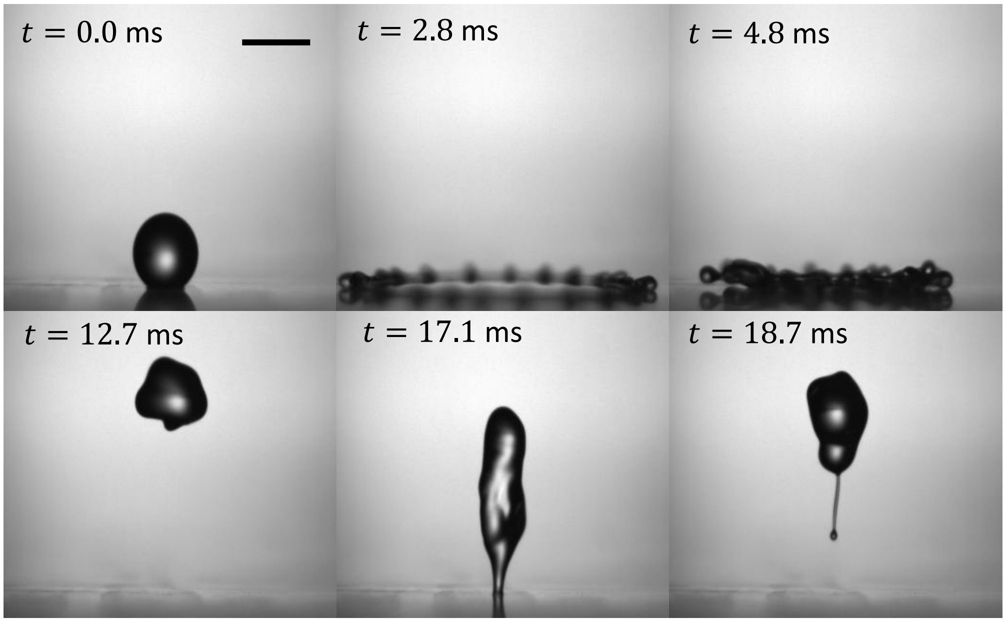

Fig. S4. Droplet of PAM 0.1% impacting a superhydrophobic surface at Weber number $We = 340$. Scale bar represents 2.5 mm.

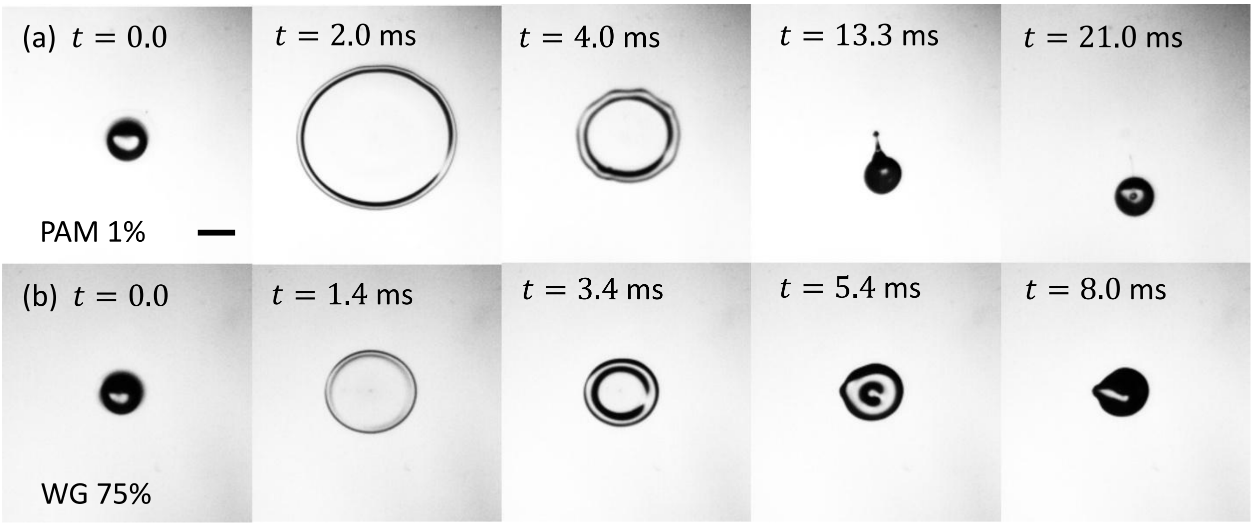

Fig. S5. (a) Droplet of PAM 1% impacting the superhydrophobic surface. (b) Water -glycerol (WG) droplet at 75% concentration. Both cases show a toroidal like shape at the maximum spreading, which suggests the suppression of fingering instability mainly by viscous effects. Impact speed is 3.7 m/s. Scale bar represents 2.5 mm.

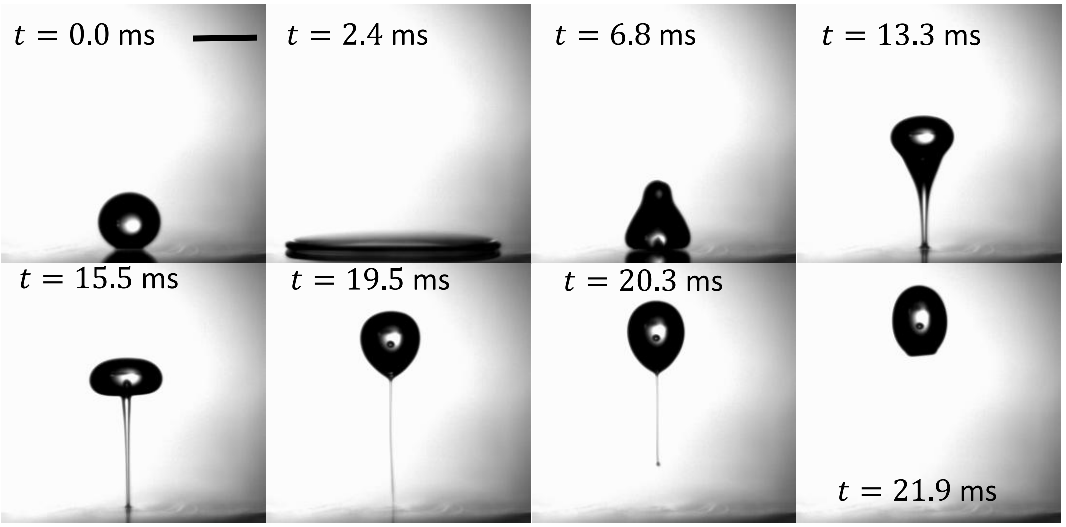

Fig. S6. Droplet of 1% PAM impacting a superhydrophobic surface at Weber number $We = 272$. Scale bar represents 2.5 mm.

VII.- Spreading and retraction dynamics of viscoelastic drops

VII.I.- Spreading phase

To describe the drop spreading dynamics, we used the well-known spreading parameter $\beta_{max} = R_{max}/R_0$, where R_{max} is the maximum contact line radius (measured from bottom view

images) and R_0 the droplet radius. scales with Weber number as $\beta{\sim}We^{1/3}$ only for Newtonian cases (water and glycerol).

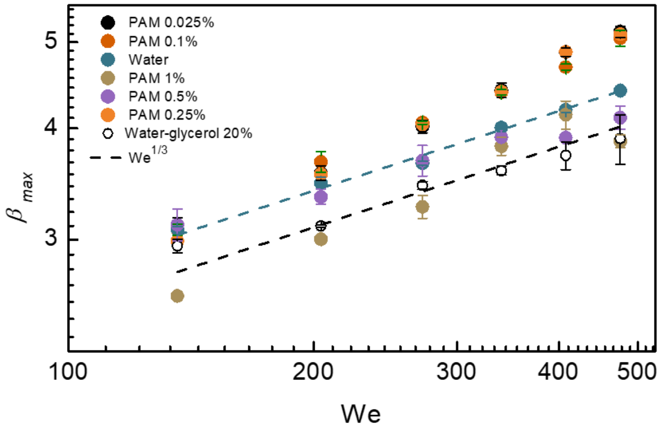

Fig. S7. Spreading parameter as a function of Weber number

VII.II Retraction phase

Plotting the spreading parameter over time for water and the non-Newtonian cases exhibiting where fingering instability revealed that all the curves collapsed onto a single curve during the receding phase. This indicates that these cases have the same retraction rate V_{ret}/R_{max} . The spreading radius was determined by fitting a circle in bottom side images, until a time lapse where the rim was no longer distinguishable.

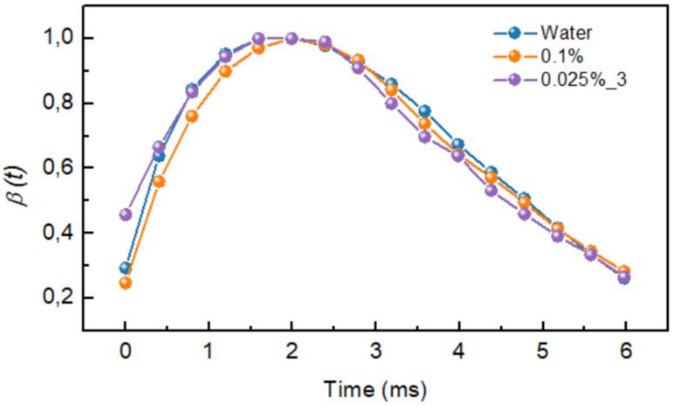

Fig. S8. Spreading parameter as a function of time for water, PAM 0.025% and PAM 0.1%

VIII.- Steady shear characterization of PAM solution with L-PTT model

For the theoretical modelling, we use the linear Phan–Thien–Tanner (L-PTT) constitutive model to track the evolution of the polymeric stress. We consider PAM concentrations of 0.025 wt% and 0.1 wt%. The overlap concentration for this polymer is c∗=0.0225%[I], giving dimensionless concentrations c/c∗=1.11 and 4.44, respectively. Both solutions therefore lie in the semi-dilute regime.

Although the L-PTT model was originally formulated for dilute polymer solutions, we use it here to provide an indicative description of polymer-stress effects on the growth of an assumed axisymmetric ligament of uniform width. The L-PTT model has also been used in our previous numerical simulations of 1 wt% PAM drop impact on a superhydrophobic surface[39], and was adopted by Sousa et al. (2011)[II] to simulate PAM solution dynamics at 0.06 wt%.

In the L-PTT model, the destruction function is approximated as a linear function of the trace of the stress tensor resulting in the following evolution equation for polymer stress tensor τ_p:

$$\left(1+\frac{\alpha\lambda}{\mu_p}tr(\tau_p)\right)\tau_p \ + \ \lambda\hat{\tau}_p = 2\mu_p D \tag{1}$$

where α is the extensibility parameter which governs the sensitivity of the relaxation time to the stress level, μ_p is the polymer viscosity, λ a characteristic time scale (equivalent to λ_{CY}) and D is the deformation rate tensor. The governing equations for the considered rheological model (L-PTT) are solved for the Couette flow configuration (steady, homogeneous simple-shear) at different shear rates and steady-shear stress response is computed using a batched damped-Newton solver. The set of parameters that fit the experimental measurements are calibrated by least-squares fitting.

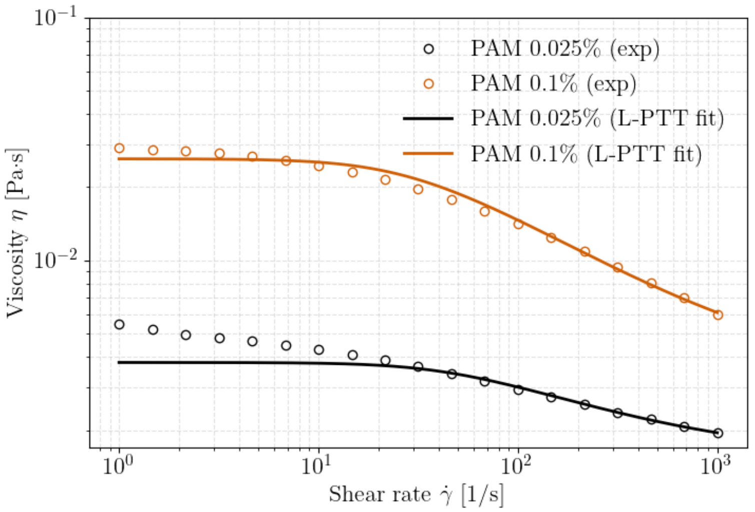

Fig. S9. The steady shear curves obtained for L-PTT model with PAM 0.025 % and PAM 0.1 %.

Table S2. Fitting parameters of L-PPT model

Parameters	PAM 0.025 %	PAM 0.1 %
λ [s]	0.075	0.11
α [-]	0.01	0.025
μ_p [Pa.s]	0.0022	0.023
$\beta = \mu_s/\mu$	0.4	0.12

It should be noted that the elastic modulus G as evaluated from the L-PTT model which is fit on the shear rheology data is < 1 Pa for both PAM 0.025 % and 0.1 %.

References:

[I] Poole, R. J. (2016). Elastic instabilities in parallel shear flows of a viscoelastic shear-thinning liquid. *Phys. Rev. Fluids*, *1*(4), 041301.

[II] Sousa, P. C., Coelho, P. M., Oliveira, M. S. N., & Alves, M. A. (2011). Effect of the contraction ratio upon viscoelastic fluid flow in three-dimensional square–square contractions. *Chemical Engineering Science*, *66*(5), 998-1009.
